# A Comprehensive Profiling of the Rice *LATERAL ORGAN BOUNDARIES DOMAIN* (*LBD*) Gene Family: Structure, Evolution, and Expressional Dynamics

**DOI:** 10.3390/plants14233596

**Published:** 2025-11-25

**Authors:** Waseem Abbas, Munsif Ali Shad, Wei Li, Abdullah Shalmani, Jian Zhang, Adnan Iqbal, Lin Liu

**Affiliations:** 1 Guangdong Provincial Key Laboratory for Plant Epigenetics, Longhua Bioindustry and Innovation Research Institute, College of Life Sciences and Oceanography, Shenzhen University, Shenzhen 518060, China; waseem@szu.edu.cn; 2 National Key Laboratory of Crop Genetic Improvement and National Centre of Plant Gene Research (Wuhan), Huazhong Agricultural University, Wuhan 430070, China; 3 Research Center for Genetic Engineering, National Research and Innovation Agency, Jakarta Pusat 10340, Indonesia; 4 Plant Breeding and Acclimatization Institute, National Research Institute, Radzikow, 05-870 Blonie, Poland

**Keywords:** rice, *Lateral Organ Boundaries Domain* transcription factor, phylogenetic analysis, expression profiling, gene duplication

## Abstract

The *LATERAL ORGAN BOUNDARIES DOMAIN* (*LBD*) gene family encodes plant-specific transcription factors that play vital roles in plant growth, development, and stress responses. Rice (*Oryza sativa* L.), a staple food for more than half of the world’s population, also serves as an important model organism for monocot functional genomics. In this study, we conducted a comprehensive genomic survey of the *OsLBD* gene family in *Oryza sativa* ssp. *japonica* using the latest genomic sequence data. A total of 35 members of this family were identified through systematic characterization of their gene structures, conserved domains, phylogenetic relationships, and chromosomal distributions. Our analysis indicated that the expansion of *OsLBD* genes may have resulted mainly from segmental duplication, with these duplicated genes exhibiting diverse evolutionary fates ranging from functional conservation to expression divergence. Phylogenetic analysis further classified the *OsLBD* genes into two major groups: Class I and Class II. Expression profiling across various developmental stages demonstrated dynamic spatiotemporal regulation, with certain genes exhibiting tissue-specific expression, particularly in reproductive tissues. Furthermore, a comprehensive co-expression analysis of *OsLBD* genes and their co-regulators revealed multiple modules with tissue-specific roles in pollen cell wall synthesis and endosperm glycogen biosynthesis. Promoter analysis identified several *cis*-regulatory elements associated with hormone responses, stress adaptation, and developmental processes, consistent with the observed expression patterns under phytohormone treatments. Comparative genomics revealed a higher degree of synteny between rice and barley than between rice and *Arabidopsis*, highlighting the evolutionary conservation within the Poaceae family. This study provides a foundational framework for understanding the biological functions of *OsLBD* genes in rice and identifies promising candidate genes involved in vegetative and reproductive growth, development, and stress responses.

## 1. Introduction

Rice is one of the most important cereal crops worldwide, consumed by nearly 50% of the global population [1]. It contributes approximately 8% of the total caloric intake of humans [2,3]. To meet the demands of a growing population, it is essential to enhance rice production and quality by investigating the molecular and genetic mechanisms underlying its growth, development, and productivity. Transcription factors play vital roles in numerous biological and cellular processes by regulating the expression of downstream genes. Among them, the *LATERAL ORGAN BOUNDARIES DOMAIN* (LBD) transcription factor family has emerged as a key regulator of plant growth, development, and stress responses. This plant-specific gene family, conserved from charophyte algae to angiosperms, plays multifaceted roles in various biological processes through its characteristic LOB/AS2 domain [4]. LBD proteins act as key regulators of diverse physiological processes across plant species, exhibiting remarkable functional versatility in both developmental programming and environmental adaptation. Beyond their well-established role in lateral organ development, these transcription factors govern a wide range of biological functions, including anthocyanin biosynthesis and nitrogen metabolism regulation in *Arabidopsis*, adventitious root formation and glume development in rice (*Oryza sativa*), as well as embryo sac differentiation and leaf morphogenesis in maize (*Zea mays*). This functional diversification underscores the evolutionary importance of LBD proteins as master regulators of plant growth and development [5,6,7,8,9,10,11].

The *LBD* gene family was first identified in *Arabidopsis* through the characterization of AtLOB (LATERAL ORGAN BOUNDARIES), which contains the defining LOB domain, also known as the AS2 (ASYMMETRIC LEAVES2) domain [12]. Subsequent studies have revealed the presence of *LBD* genes across a wide range of plant species, including 43 members in *Arabidopsis* [13], 44 in *Zea mays* [14], 131 in *Gossypium hirsutum* [15], 90 in *Triticum aestivum* [16], and 59 in *Brassica rapa var. rapa* [17]. LBD proteins generally consist of three conserved regions: first, an N-terminal domain featuring a zinc-finger-like motif (CX_2_CX_6_CX_3_C) that is essential for DNA binding; second, a central GAS (Gly-Ala-Ser) block containing functionally important residues—a conserved proline within the GAS block of the *AtLBD18* LOB domain is critical for both DNA-binding activity and biological function in *Arabidopsis*; and third, a leucine-zipper motif (LX_6_LX_3_LX_6_L) of approximately 30 amino acids, which facilitates protein dimerization and molecular interactions through its amphipathic α-helical structure. This conserved motif, located adjacent to the DNA-binding domain, facilitates the formation of functional transcription factor complexes essential for regulatory activity [5,18]. The evolutionarily divergent C-terminal domain acts as a molecular switch, with its sequence variability conferring either transcriptional activation or repression functions through differential interactions with chromatin remodelers, basal transcription machinery, and other regulatory complexes [9]. Based on structural characteristics, LBD proteins are classified into two major classes: Class I, which forms coiled-coil structures and is further divided into four subclasses (IA, IB, IC, and IE), and Class II, which lacks intact leucine-zipper domains and consists of two subclasses (IIA and IIB) [5,11,19].

Functionally, *LBD* genes are involved in a wide range of developmental processes and stress responses across plant species. In *Arabidopsis*, *AS2* regulates leaf lamina expansion and vein patterning [20], while *DDA1* (*DOWN IN DARK AND AUXIN1*) plays a role in auxin signaling and photomorphogenesis [21]. Rice orthologs exhibit similarly diverse functions; for example, *OsAS2* contributes to leaf differentiation [22], while *OsLBD37, OsLBD38*, and *OsLBD39* regulate nitrate uptake in response to nitrogen availability [23]. Despite previous research, significant gaps remain in our understanding of the complex regulatory networks governing the functions of *OsLBD* genes in plant growth and development. To address these gaps, we conducted a comprehensive genomic survey and expression profiling analysis of the *OsLBD* gene family in *Oryza sativa subsp. japonica.* Our study includes gene structure analysis, conserved domain characterization, phylogenetic reconstruction, chromosomal localization, collinearity assessment, duplication event analysis, and *cis*-regulatory element identification. Furthermore, we provided detailed expression profiles of *OsLBD* genes across various developmental stages of rice, including vegetative growth, flowering transition, and grain-filling phases. We also examined their tissue-specific regulation and transcriptional responses to different hormone treatments and environmental stress conditions. Moreover, we identified co-expressed gene modules with tissue-specific functions, including pollen cell wall synthesis and endosperm glycogen biosynthesis, highlighting the roles of *OsLBD* genes in reproductive development. This systematic study not only provides a foundational framework for understanding the evolution and function of *OsLBD* genes in rice but also highlights potential targets for the genetic improvement of agronomic traits. By elucidating the molecular mechanisms underlying *OsLBD*-mediated regulation, our work contributes to ongoing efforts to enhance rice productivity and stress resilience, offering valuable insights for future crop improvement strategies.

## 2. Results

### 2.1. Genome-Wide Identification and Characterization of OsLBD Genes

Our genome-wide analysis identified 35 *OsLBD* genes in *Oryza sativa ssp. japonica* genome, which represents an expansion from the 31 members previously reported [24]. A direct comparison revealed that our list includes four genes (LOC_Os01g39150, LOC_Os01g39160, LOC_Os01g39180 and LOC_Os01g39220) that were missing in the previous study, thereby providing a more comprehensive genomic survey [24]. Phylogenetic classification based on the presence of the characteristic leucine zipper-like motif (LX_6_LX_3_LX_6_L) revealed clear evolutionary divergence, with 30 members assigned to Class I (possessing intact zipper domains) and five members to Class II (lacking complete zipper architecture). Comprehensive biophysical characterization using ExPASY revealed substantial variation among the OsLBD proteins, with lengths ranging from 125 amino acids (LOC_Os01g39070) to 456 amino acids (LOC_Os08g31080) and corresponding molecular weights between 13.3 and 48.7 kDa. The predicted isoelectric points ranged from acidic to basic (pI 4.62–9.72), indicating potential functional diversification in charge-mediated interactions. Stability analysis suggested that most proteins are predicted to be unstable, with the exceptions of LOC_Os08g31080 and LOC_Os01g66590 (Table 1) [25].

The aliphatic indices (54.49–85.86) indicate relatively conserved thermostability among the family members, while the negative grand average of hydropathicity (GRAVY) scores (−0.88 to 0.166) confirm their predominantly hydrophilic nature. Consistent with their proposed roles as transcription factors, all 35 OsLBD proteins are predicted to localize in the nucleus.

### 2.2. Chromosomal Distribution and Multiple Sequence Alignment of OsLBD Genes

**Figure 1:**
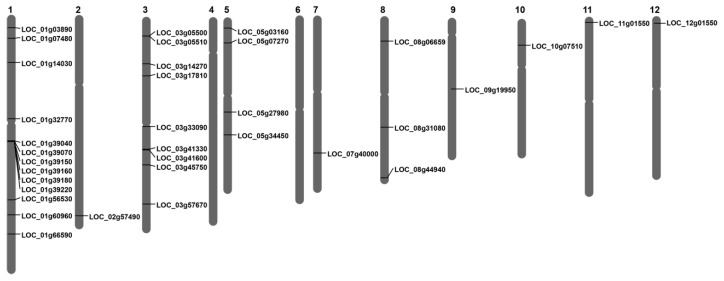
Chromosomal distribution of *OsLBDs* genes in rice. Black lines on chromosomes represent the location of each *OsLBD* gene. Chromosome numbers are indicated above each chromosome. Chromosomes 4 and 6 contain no *OsLBD* gene.

Using the Oryzabase genomic resource (https://shigen.nig.ac.jp/rice/oryzabase/, accessed on 1 December 2024), we mapped all 35 identified *OsLBD* genes across the *Oryza sativa* ssp. *japonica* genome. The genes were unevenly distributed across 10 of the 12 chromosomes, with no members found on chromosomes 4 and 6. Chromosomes 1 and 3 were identified as major hotspots, containing 13 and 9 OsLBD genes, respectively, representing 37% and 26% of the total gene family. The remaining genes were located singly on chromosomes 2, 7, 9, 10, 11, and 12. Notably, chromosome 1 harbored a prominent gene cluster of six tandemly arrayed OsLBD genes (LOC_Os01g39040, LOC_Os01g39070, LOC_Os01g39150, LOC_Os01g39160, LOC_Os01g39180, and LOC_Os01g39220) within a 200 kb region, while chromosome 3 contained two pairs (LOC_Os03g5500/LOC_Os03g5510 and LOC_Os03g41330/LOC_Os03g41600) (Figure 1). This uneven genomic distribution suggests that chromosomal rearrangements may have been a key mechanism in the expansion of the *OsLBD* gene family in rice.

**Figure 2:**
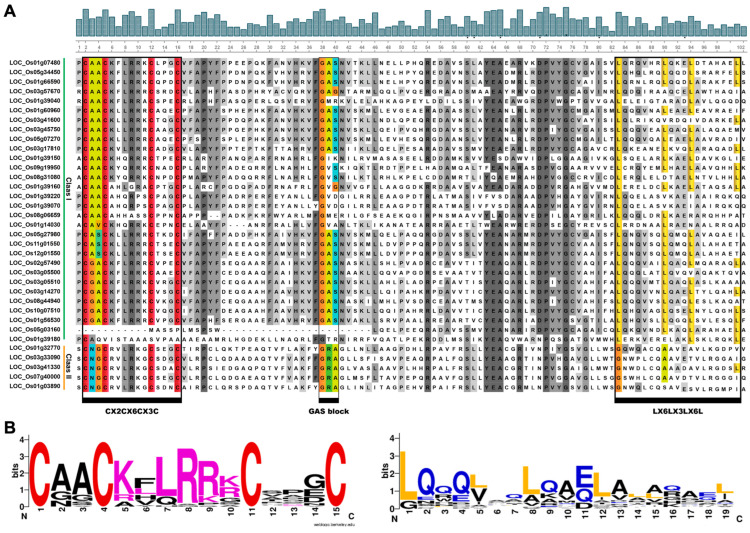
Multiple sequence alignment of the conserved domain of the OsLBD protein in rice. (**A**) Multiple sequence alignment showing the CX_2_CX_6_CX_3_C zinc finger-like domain (present almost in all OsLBD proteins) and the leucine zipper-like motif (LX_6_LX_3_LX_6_L, exclusive to Class I). (**B**) Sequence logos of conserved motifs generated using WebLogo V2.

We analyzed the conserved domains of all 35 OsLBD proteins by performing a multiple sequence alignment of their LOB domains (CX_2_CX_6_CX_3_C) using ClustalW and visualizing conserved motifs with WebLogo. The analysis revealed that nearly all OsLBD family members possess a highly conserved ~100 amino acid LOB region at the N-terminus, which includes the characteristic zinc finger domain. Among these, 30 OsLBD members (85.72%) belong to Class I, while the remaining five members (14.28%) are classified as Class II. The CX_2_CX_6_CX_3_C zinc finger-like domain was present in all sequences except for LOC_Os05g03160, where complete absence is observed, and LOC_Os01g39180, which shows partial loss. Furthermore, the leucine zipper-like domain (LX_6_LX_3_LX_6_L), despite some sequence variation, was found exclusively in Class I OsLBD proteins, consistent with observations in other plant species (Figure 2) [15,16]. These conserved domain features highlight both the evolutionary preservation of key structural elements across the OsLBD family and the distinct characteristics that differentiate Class I and Class II members.

### 2.3. Phylogenetic Analysis and Evolutionary Relationships of OsLBDs

**Figure 3:**
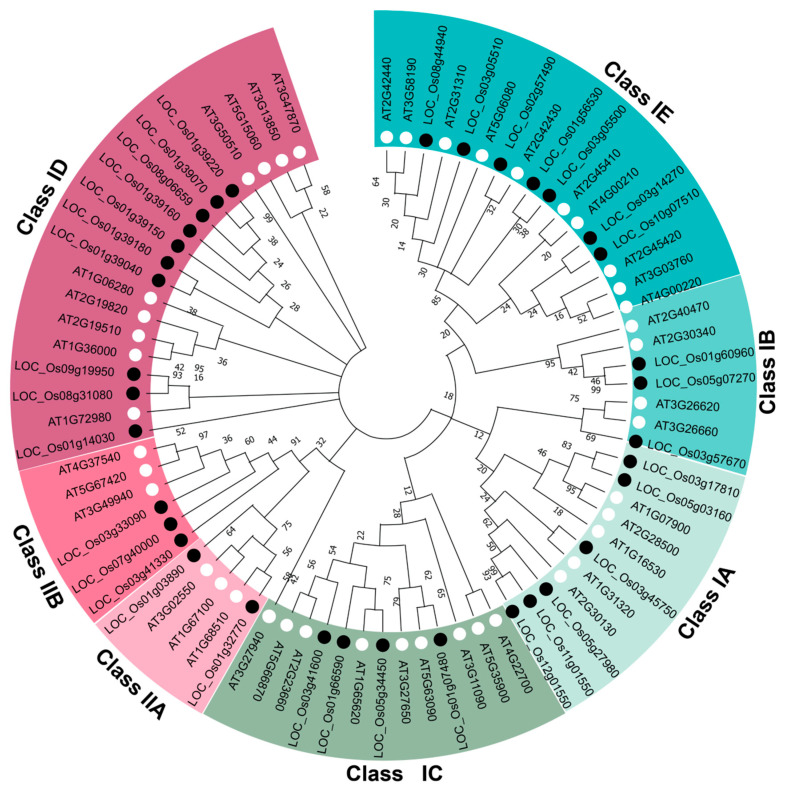
Phylogenetic analysis of OsLBD proteins in *Oryza sativa* and *Arabidopsis thaliana*. The evolutionary tree was constructed using the maximum likelihood (ML) method in MEGA 11 with 1000 bootstrap replicates. Rice and *Arabidopsis* LBD proteins are marked with black and white circles, respectively. Different colored branches represent distinct classes and subclasses.

To examine the evolutionary conservation of LBD transcription factors, we constructed a maximum likelihood phylogenetic tree using the full-length amino acid sequences of 35 rice (OsLBD) and 43 *Arabidopsis* (AtLBD) proteins. The analysis identified two well-supported major classes (I and II) conserved across both species. Class I, which represents the majority of genes, was further subdivided into five distinct subclasses: IA, containing 6 *OsLBD* and 5 *AtLBD* genes; IB, with 3 *OsLBD* and 4 *AtLBD* members; IC, comprising 4 *OsLBD* and 9 *AtLBD* genes; ID, consisting of 10 *OsLBD* and *9 AtLBD* representatives; and IE, encompassing 7 *OsLBD* and 10 *AtLBD* sequences. The smaller Class II is divided into two subclasses: IIA, containing 2 *OsLBD* and 3 *AtLBD* genes, and IIB, comprising 3 *OsLBD* and 3 *AtLBD* members (Figure 3). This conserved phylogenetic structure, despite the divergent evolution of monocots and dicots, suggests that core *LBD* gene functions have been preserved while allowing for lineage-specific expansions within certain subclades, potentially reflecting functional diversification. Strong bootstrap support at all major nodes indicates robust evolutionary relationships among these transcription factor families.

### 2.4. Gene Structure and Conserved Motif Analysis of the Rice OsLBD Gene Family

**Figure 4:**
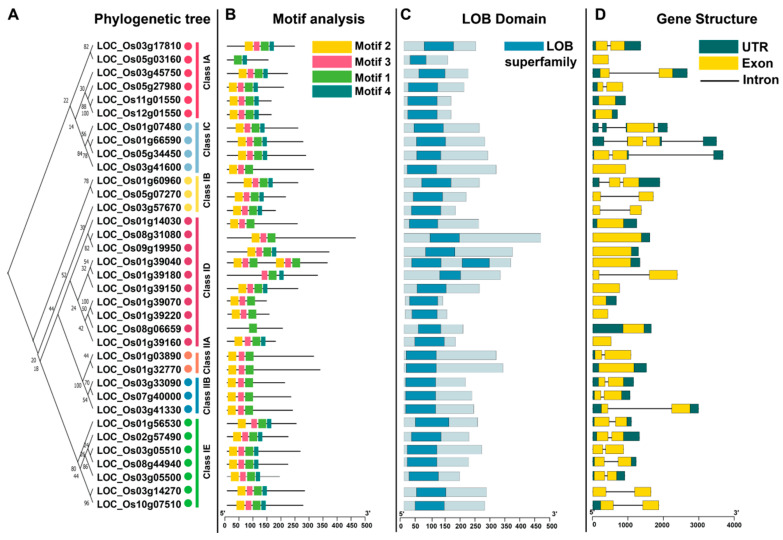
Phylogenetic, conserved motif, and gene structure analysis of OsLBD proteins. (**A**) Maximum likelihood (ML) phylogenetic tree constructed using full-length OsLBD protein sequences (1000 bootstrap replicates). (**B**) Distribution of conserved motifs in OsLBD proteins (scale bar = 50 amino acids). (**C**) Localization of the LOB domain within *OsLBD* genes. (**D**) Gene structure of *OsLBD* genes, illustrating introns (black lines), exons (yellow rectangles), and untranslated regions (UTRs, green rectangles; scale bar = 1 kb).

To further clarify the evolutionary relationships and structural organization of rice *LBD* genes, we conducted comprehensive phylogenetic analyses. The reconstruction strongly supported the division of *OsLBD* genes into two major classes: Class I, comprising five subclasses (IA, IB, IC, ID, and IE, with 6, 3, 4, 10, and 7 members, respectively), and Class II, containing two subclasses (IIA and IIB, with 2 and 3 members, respectively (Figure 4A). This classification was further validated through detailed analysis of conserved protein motifs and gene structures. Conserved motif analysis identified four core motifs (Motif 1–Motif 4) that characterize the OsLBD protein family. Motif 1, found in all OsLBD members, contains the characteristic CX_2_CX_6_CX_3_C zinc finger-like domain, which is essential for DNA binding. Motifs 2 and 3, forming the conserved portion of the LOB domain, are present in all members except LOC_Os05g03160, LOC_Os01g39180, and LOC_Os08g06659, suggesting potential functional specialization in these variants. Notably, Motif 4 contains the LX6LX3LX6L leucine zipper-like domain and aligns perfectly with the phylogenetic classification, being exclusive to Class I members and absent in Class II. This distinct pattern not only validates our classification system but also indicates that the presence or absence of this dimerization domain represents a fundamental evolutionary divergence within the *OsLBD* family (Figure 4B,C). Gene structure analysis revealed that *OsLBD* family members generally exhibit simple genomic architectures, with 21 genes containing two exons and 14 genes consisting of a single exon. Notably, seven genes have intron sequences longer than their exons, suggesting potential regulatory complexity. Within phylogenetic subclasses, exon–intron patterns are highly conserved. Sixteen members across subclasses IC, IB, IIB, and IE consistently display a two-exon structure, with the exceptions of LOC_Os03g41600 and LOC_Os01g07480, which contain a single exon. Similarly, the ID subclass shows remarkable uniformity, with all nine members having a single exon except for LOC_Os01g39180, which contains two exons. In contrast, subclasses IA and IIB exhibit greater structural heterogeneity, with exon numbers ranging from one to two among members, indicating possible functional diversification within these groups (Figure 4D).

### 2.5. Cis-Regulatory Element Analysis in OsLBD Promoters

**Figure 5:**
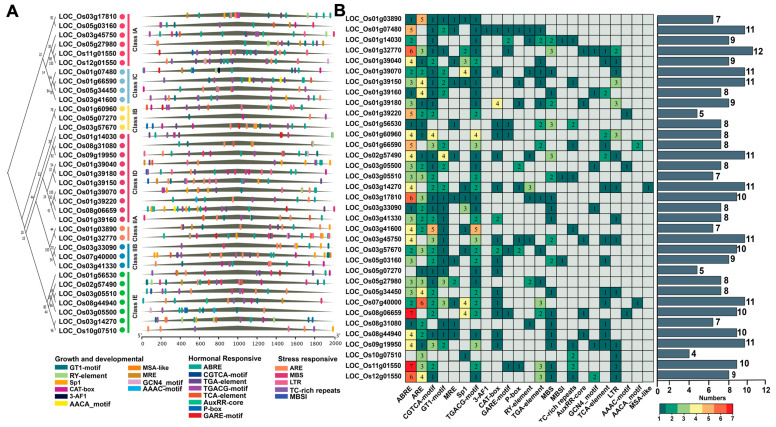
Analysis of *cis*-elements in *OsLBD* promoters. (**A**) Distribution of *cis*-elements identified within the 2000 bp upstream promoter regions of *OsLBD* genes. (**B**) Numbers of *cis*-elements present in the putative promoter regions of *OsLBD* genes.

*Cis*-elements are DNA sequences that regulate gene expression without encoding proteins or RNA. These non-coding sequences, typically located in promoter regions, control the transcription of their associated genes. In this study, we analyzed the 2000 bp region upstream of the transcription start site of each *OsLBD* gene as the putative promoter. Using the PlantCARE database, we identified a diverse set of 23 representative *cis*-regulatory elements (Figure 5A,B and Table S1a,b). In addition to core promoter elements, numerous functional motifs were detected, including those responsive to light (GT1-motif, 3-AF1 binding site, Sp1), low temperature (LTR), defense and stress (TC-rich repeats), and anaerobic conditions (ARE). We also identified MYB binding sites (MBS, MBSI) associated with drought response and flavonoid biosynthesis, along with hormone-responsive elements for salicylic acid (TCA-element), methyl jasmonate (CGTCA-motif, TGACG-motif), auxin (TGA-element), abscisic acid (ABRE), and gibberellin (P-box, TATC-box, GARE-motif). Additionally, elements involved in tissue-specific expression (RY-element, CAT-box) and developmental processes (MSA-like, circadian elements) were detected. Among the identified cis-elements, ABRE motifs were the most abundant (116), followed by ARE motifs (71) and methyl jasmonate-responsive elements (56 TGACG-motifs and 56 CGTCA-motifs), which were distributed across both Class I and Class II *OsLBD* promoters (Figure 5A). Notably, each gene contained equal numbers of TGACG and CGTCA motifs; for example, LOC_Os03g41600 contained five of each, while LOC_Os01g66590 had three of each. MYB binding sites were present in all subclasses except IB and IC, while the light-responsive 3-AF1 element was uniquely found in LOC_Os01g07480 (Figure 5B). Collectively, these results indicate that the functional expression of *OsLBD* genes in rice is regulated by a complex network of cis-acting elements that mediate hormone responses, coordinate growth and developmental processes, and contribute to stress adaptation.

### 2.6. Expression of OsLBD Genes Under NAA, KT, and GA3 Hormonal Treatments

**Figure 6:**
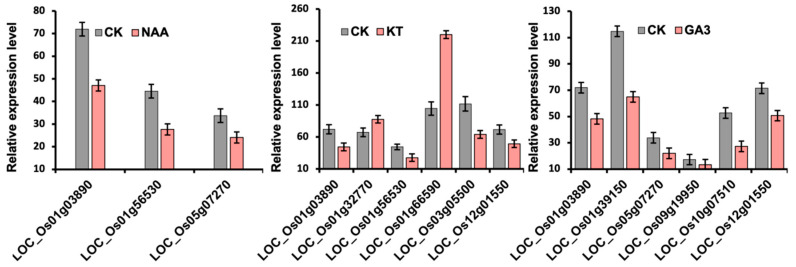
Differential expression of *OsLBD* genes in rice seedlings in response to phytohormone treatments (NAA, KT, and GA3). Expression levels are shown relative to the untreated control (CK). Values represent average microarray expression scores from two biological replicates.

Expression analysis of *OsLBD* genes in rice seedlings in response to phytohormone treatments revealed that 10 *OsLBDs* exhibited significant differential expression under NAA (auxin), KT (cytokinin), or GA3 (gibberellin) compared with untreated controls (Figure 6 and Table S2). LOC_Os01g66590 was specifically upregulated under KT treatment, suggesting a positive role in cytokinin signaling, whereas most responsive genes were downregulated. Under KT treatment, four genes (LOC_Os01g03890, LOC_Os12g01550, LOC_Os01g56530, and LOC_Os03g05500) were downregulated, while two genes (LOC_Os01g32770 and LOC_Os01g66590) were upregulated. Under GA3 treatment, six genes (LOC_Os01g03890, LOC_Os01g39150, LOC_Os05g07270, LOC_Os09g19950, LOC_Os10g07510, and LOC_Os12g01550) were downregulated. In response to NAA treatment, three genes (LOC_Os01g03890, LOC_Os01g56530, and LOC_Os05g07270) showed downregulation. Notably, LOC_Os01g03890 exhibited consistent downregulation across all three phytohormone treatments, suggesting a potential negative role in hormone signaling pathways. These findings indicate that *OsLBD* genes are differentially regulated by multiple phytohormones, with most being downregulated, which suggests that they may act as negative regulators in these signaling pathways, while LOC_Os01g66590 may serve as a positive regulator in cytokinin-mediated processes.

Moreover, the observed expression profiles of *OsLBD* genes partially correspond with the predicted *cis*-regulatory elements (Figure 5 and Table S1). Specifically, five genes (LOC_Os01g03890, LOC_Os01g32770, LOC_Os01g66590, LOC_Os03g05500, and LOC_Os12g01550) contain cytokinin (KT)-responsive motifs in their promoter regions, including CGTCA, TGACG, and AAAC motifs. In contrast, four genes (LOC_Os01g39150, LOC_Os05g07270, LOC_Os09g19950, and LOC_Os12g01550) possess gibberellin (GA3)-responsive elements, such as the CAT-box, P-box, and TGA-element. Notably, LOC_Os01g56530 contains an auxin (NAA)-responsive TGA-element in its promoter region (Figure 5). Overall, these findings suggest that the presence of hormone-specific *cis*-elements may contribute to the differential expression patterns observed under phytohormone treatments.

### 2.7. OsLBD Gene Duplication and Expression Divergence

**Figure 7:**
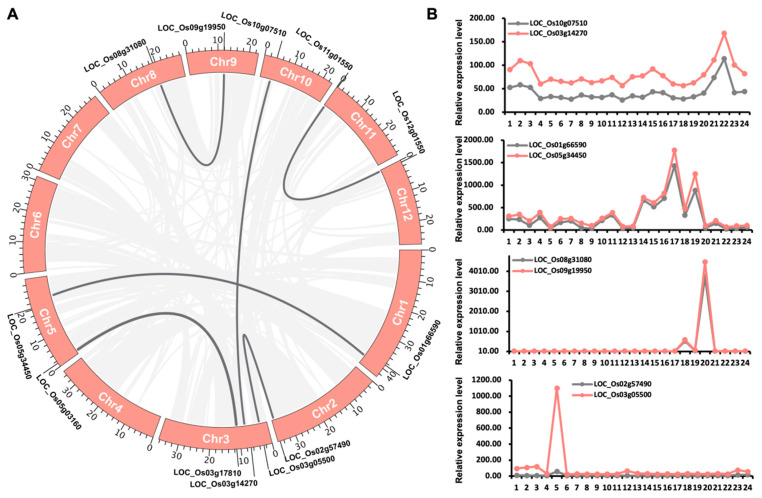
Gene duplication and expression patterns of *OsLBD* genes. (**A**) Gene duplication events among *OsLBD* genes. Gray background lines indicate collinear regions, while dark gray lines highlight collinear pairs of *OsLBD* members. (**B**) Expression profiles of duplicated gene pairs across developmental stages. The *X*-axis represents different developmental stages, while the *Y*-axis shows raw microarray expression values. Sample and expression details are provided in Tables S3 and S4.

Both segmental and tandem duplication events are key drivers of gene family expansion and species diversification. To explore the mechanisms underlying the evolution of the *OsLBD* gene family, we analyzed both types of duplication. Our results identified twelve segmentally duplicated *OsLBD* genes distributed across nine rice chromosomes, with no duplicates on chromosomes 4, 6, or 7. Chromosome 3 contained the highest number of duplicated genes (three), while the remaining chromosomes each harbored a single duplicated *OsLBD* gene. Notably, all detected duplication events were segmental, with no evidence of tandem duplication within this gene family (Figure 7A).

Furthermore, our analysis revealed phylogenetic conservation of gene duplication events within the *OsLBD* family. All duplicated gene pairs were confined to Class I, with each pair belonging to the same subclass: 1A (LOC_Os03g17810, LOC_Os05g03160, LOC_Os12g01550, LOC_Os11g01550), 1C (LOC_Os05g34450, LOC_Os01g66590), 1D (LOC_Os08g31080, LOC_Os09g19950), and 1E (LOC_Os02g57490, LOC_Os03g05500, LOC_Os03g14270, LOC_Os10g07510). No duplication events were observed among Class II members. The complete absence of duplications in Class II highlights a fundamental evolutionary divergence between the two major clades, suggesting distinct selective pressures that have shaped their genomic stability and expansion patterns.

In addition, we analyzed the expression patterns of duplicated *OsLBD* gene pairs using available Affymetrix microarray probe sets, which covered 4 of the 6 duplicated pairs (8 genes in total). Among these, three gene pairs displayed nearly the same expression profiles, suggesting conserved functions following duplication. However, one pair (LOC_Os03g14270/LOC_Os10g07510) exhibited differential expression levels, with LOC_Os03g14270 showing higher expression while retaining a similar overall pattern. Notably, the LOC_Os08g31080/LOC_Os09g19950 pair demonstrated strong and specific co-expression in stamen tissue, with minimal expression detected in all other tissues. Most notably, the LOC_Os03g05500/LOC_Os02g57490 pair exhibited complete expression divergence, with LOC_Os03g05500 showing root-specific expression and LOC_Os02g57490 being nearly transcriptionally inactive across all tissues (Figure 7B). These patterns illustrate the variable evolutionary fates of duplicated genes, ranging from retained redundancy (most pairs) to potential subfunctionalization (stamen-specific pair), or in some cases, where one duplicate retains function while its duplicate (LOC_Os02g57490) appears to have lost transcriptional activity or been silenced, as no expression was detected.

### 2.8. Collinearity and Evolution Analysis of OsLBD Genes

**Figure 8:**
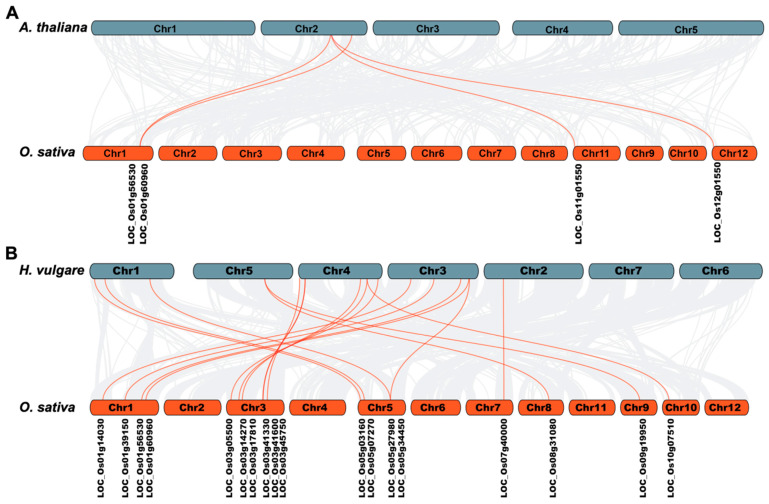
Synteny analysis of *LBD* genes among rice (*Oryza sativa*), *Arabidopsis* (*A. thaliana*, dicot), and barley (*Hordeum vulgare*, monocot). (**A**,**B**) Gray background lines represent collinear genomic blocks, while red lines highlight syntenic *LBD* gene pairs. Species abbreviations are indicated as follows: *O* (*Oryza sativa*), *A* (*Arabidopsis thaliana*), and *H* (*Hordeum vulgare*).

To explore the evolutionary relationships of *OsLBD* genes, we conducted a collinearity analysis comparing these genes with their orthologs in *Arabidopsis* (a dicot) and barley (a monocot). This comparative genomic approach provides insights into evolutionary conservation and divergence. Our analysis revealed distinct patterns of genomic conservation: four *OsLBD* genes displayed collinearity with *Arabidopsis* (Figure 8A), whereas a substantially higher number (18 *OsLBD* genes) showed collinearity with barley, indicating conserved synteny within the grass family (Poaceae) (Figure 8B). The degree of collinearity was notably higher between rice and barley than between rice and *Arabidopsis*, reflecting their closer phylogenetic relationship as members of the grass family compared with the more distantly related dicot *Arabidopsis*. These findings underscore the conserved genomic organization of *LBD* genes within the grass family and highlight the evolutionary divergence between monocots and dicots.

### 2.9. Expression Pattern of OsLBD Genes Throughout the Rice Life Cycle

**Figure 9:**
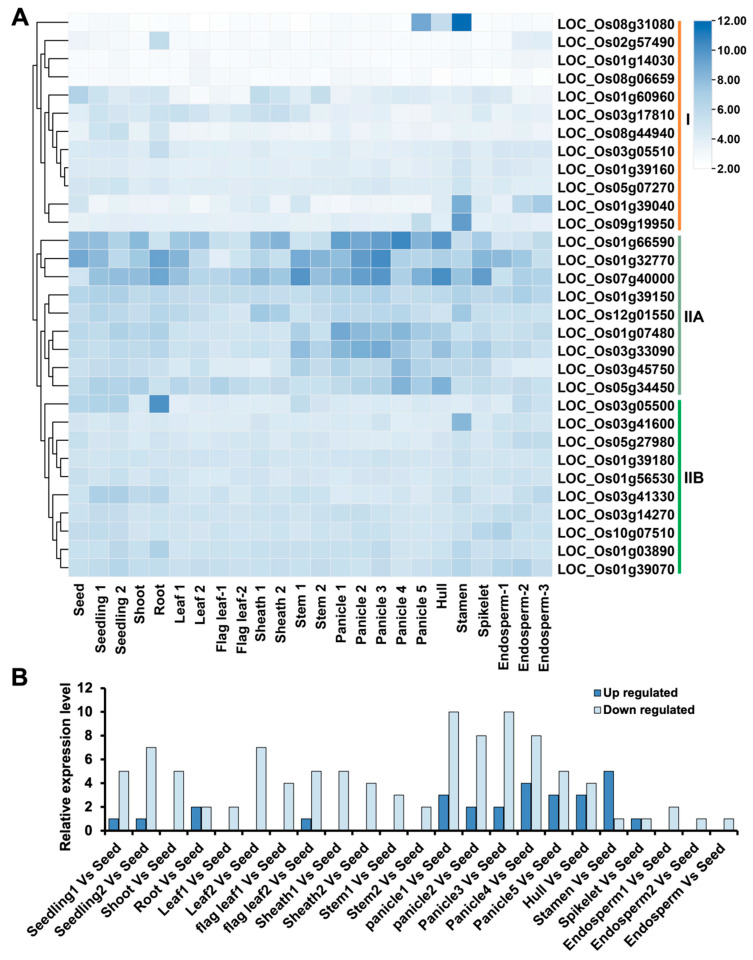
Expression patterns of *OsLBD* genes across the rice life cycle. (**A**) Hierarchical clustering of 31 *OsLBD* genes was performed using Affymetrix microarray data to analyze their expression profiles. The color gradient (dark blue = high, light blue = moderate, white = low) represents log2-transformed expression values. (**B**) Stage-specific expression profiles of *OsLBD* genes in Minghui 63 rice cultivar. Detailed information on the developmental stages is provided in Tables S3–S5.

Our analysis of Affymetrix microarray data from the CREP database revealed distinct expression profiles of *OsLBD* genes across 24 developmental stages in Minghui 63 rice. Probes were identified for 31 of the 35 *OsLBD* genes; however, four genes (LOC_Os01g39220, LOC_Os03g57670, LOC_Os05g03160, and LOC_Os11g01550) lacked corresponding probes on the array platform. For genes with multiple probe sets, the probe exhibiting the strongest signal was selected to ensure reliable data representation. Hierarchical clustering of the expression patterns divided the 31 genes into two major groups: Group I contained 12 genes that generally showed low expression across all tissues, with the notable exception of LOC_Os08g31080, which displayed higher expression in hull and spikelet tissues. Group II comprised 19 genes with significantly increased expression levels, which were further subdivided into two subgroups. In subgroup IIA, LOC_Os01g66590, LOC_Os01g32770, LOC_Os07g40000, LOC_Os01g07480, and LOC_Os03g33090 exhibited particularly strong expression throughout the rice life cycle, peaking during reproductive stages in panicles, hulls, and spikelets. Subgroup IIB included 14 genes that maintained moderate but consistent expression across all developmental stages. These distinct expression patterns suggest specialized functional roles for different *OsLBD* members. Genes in subgroup IIA are likely important for reproductive development, whereas subgroup IIB genes may contribute to general growth processes. Most Group I genes appear to function as fine-tuned regulators, with the exception of LOC_Os08g31080, which seems to have acquired a specialized role in floral tissues (Figure 9A).

To investigate the developmental regulation of *OsLBD* genes, we analyzed their differential expression across various tissues relative to seed expression (complete statistical data, including *p*-values and fold changes, are provided in Table S5). Most genes exhibited significant up- or down-regulation during panicle formation and floral organ development (hulls, stamens, and spikelets), indicating their involvement in reproductive molecular pathways. Five *OsLBD* genes (LOC_Os01g39040, LOC_Os03g41600, LOC_Os07g40000, LOC_Os08g31080 and LOC_Os09g19950) were upregulated in stamens, representing the highest proportion of upregulated genes in any tissue. Notably, LOC_Os08g31080 and LOC_Os09g19950 showed specific upregulation in panicles and stamens, suggesting specialized roles in rice reproductive development. In contrast, LOC_Os07g40000 exhibited broad activation patterns, with significant upregulation in both vegetative and reproductive tissues, suggesting a potential dual role in early seedling establishment and floral development (Figure 9B). These expression analyses reveal that *OsLBD* genes display dynamic patterns across all major developmental stages of rice, from vegetative growth to reproductive maturation. The stage-specific and tissue-preferential expression of certain *OsLBD* members—including five genes highly upregulated in pre-flowering stamens and others peaking in panicles or vegetative tissues—highlights their essential and diverse functional contributions throughout the rice life cycle.

### 2.10. Identification of Co-Expressed Genes and Gene Enrichment Analysis

**Figure 10:**
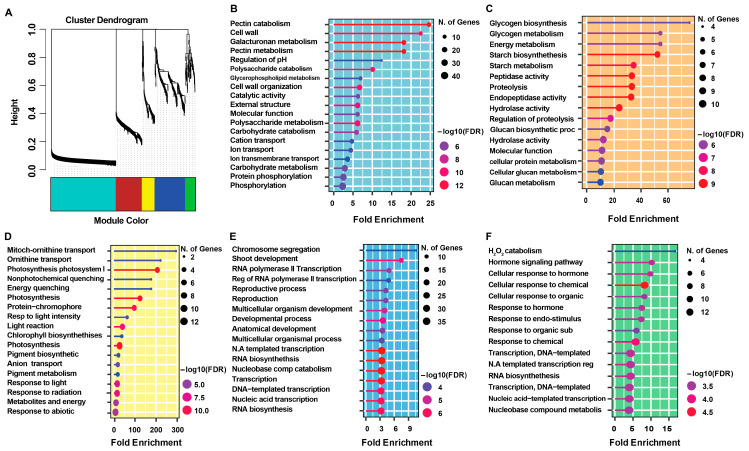
Co-expression and functional enrichment analysis of *OsLBD* genes. (**A**) Weighted Gene Co-expression Network Analysis (WGCNA) analysis showing co-expressed gene modules (colored: turquoise, brown, yellow, blue, and green). (**B**–**F**) Gene Ontology enrichment analysis of co-expressed modules using ShinyGO, with each color corresponding to its respective module. The significance of enrichement is represented by the fold enrichment relative to the background.

Co-expressed genes form co-regulated clusters that are often transcribed by common classes of transcription factors. These genes frequently encode proteins that participate in specific biological pathways [26]. In this study, the co-expression matrix included 877 genes whose expression patterns were tightly correlated with the 35 *OsLBD* family guide genes. Clustering analysis of these co-expressed genes identified five distinct modules, represented by the colors turquoise, brown, yellow, blue, and green (Figure 10).

The turquoise module was the largest, containing 409 genes, including four *OsLBD* guide genes. According to the CREP database expression data, these genes showed tissue-specific upregulation in pollen. Gene ontology analysis of this module identified 16 pectin lyase fold genes and 12 calcium-binding EF-hand domain genes, in addition to the four guide genes, suggesting a key role in cell wall synthesis during pollen development. The brown module comprised 166 co-expressed genes associated with a single guide gene. Genes in this module were preferentially upregulated in endosperm tissues and enriched for functional classes related to plant lipid transfer proteins and storage/trypsin-alpha amylase inhibitor genes, suggesting a potential role in glycogen biosynthesis during endosperm development. The yellow, blue, and green modules also showed significant enrichment in specific biological processes (Figure 10D–F). The yellow module was strongly associated with photosynthesis-related processes, with the most enriched Gene Ontology (GO) terms including Photosystem I, Energy quenching, and Chlorophyll biosynthesis. The blue module was predominantly related to transcriptional regulation and developmental processes, with key GO terms such as chromosome segregation, RNA polymerase II transcription, reproductive process, and multicellular organism development. The green module was enriched for hormone and stress-related responses. Key GO terms included H_2_O_2_ catabolism, hormone signaling pathway, and cellular response to hormone/chemical/organic stimulus. The modular organization of gene expression observed in this WGCNA underscores the functionally distinct roles of different co-expressed gene clusters. Each module contributes to specific aspects of plant growth, reproduction, and environmental adaptation, reflecting the remarkable functional diversification within the *OsLBD* co-expression network. Overall, these findings reveal biologically meaningful gene modules and provide valuable insights into the distinct regulatory and physiological roles coordinated by each module.

## 3. Materials and Methods

### 3.1. Data Acquisition and Sequence Analysis

The members of *OsLBD* gene family in *Oryza sativa subsp. Japonica* were identified and their locus RGAP IDs were collected from Plant Transcription Factor Database (http://planttfdb.gao-lab.org/ v5.0). The genome-wide protein sequences, genomic sequence and GFF annotation files (General Feature Format) for *Oryza sativa subsp. Japonica* were obtained from the Rice Genome Annotation Project Database (http://rice.plantbiology.msu.edu/), providing comprehensive gene models and functional predictions for protein-coding genes. For comparative analyses, *Arabidopsis thaliana* genomic data and 43 experimentally validated AtLBD protein sequences were retrieved from The Arabidopsis Information Resource (TAIR; https://www.arabidopsis.org), while barley (*Hordeum vulgare*) genomic sequence and annotations were acquired from *Phytozome* v13 (https://phytozome-next.jgi.doe.gov).

### 3.2. Multiple Sequence Alignment and Phylogenetic Tree Construction

Initially 36 putative OsLBD protein sequences were retrieved using the Rice Genome Annotation Project database (http://rice.plantbiology.msu.edu/). Following the removal of one redundant gene, the remaining 35 non-redundant OsLBD protein sequences were subjected to multiple sequence alignment using the MUSCLE algorithm as implemented in Unipro UGENE V.1.18.0 software [27]. Conserved sequence motifs were visualized through the online WebLogo tool V2 (http://weblogo.threeplusone.com). For comparative evolutionary analysis, we obtained the complete set of 43 experimentally validated AtLBD protein sequences from Arabidopsis Information Resource (TAIR; https://www.arabidopsis.org). Comprehensive phylogenetic analysis was conducted by first performing multiple sequence alignment of all rice and *Arabidopsis* LBD protein sequences using ClustalW with default parameters. The resulting alignment was then used to construct maximum likelihood phylogenetic trees in MEGA 11 software, with 1000 bootstrap replicates [28].

### 3.3. Cis-Regulatory Elements, Gene Structure, Conserved Motif, and Domain Analysis of OsLBD Genes

To identify putative *cis*-regulatory elements, we extracted 2 kb promoter sequences upstream of the transcription start site for each *OsLBD* gene by using TBtools (V1.0986). These sequences were analyzed using the PlantCARE web tool (https://bioinformatics.psb.ugent.be/webtools/plantcare/html/, accessed on 1 January 2025) to predict *cis*-acting regulatory elements [29]. To better understand the distribution of *cis-elements*, we manually organized the PlantCARE output in Excel and generated a heatmap of all *cis*-elements for *OsLBD* genes. Gene structure analysis was performed using the GFF annotation file obtained from the Rice Genome Annotation Project Database (http://rice.plantbiology.msu.edu/). For conserved protein motif analysis, we employed the MEME Suite (https://meme-suite.org/meme/, accessed on 1 January 2025) with optimized parameters: motif discovery set to identify up to 4 distinct motifs per sequence, with widths ranging between 6 and 100 residues. Conserved domains were further verified using the Conserved Domain Database (CDD; https://www.ncbi.nlm.nih.gov/cdd, accessed on 1 January 2025). All genomic and protein features were visualized and annotated using TBtools [30].

### 3.4. Chromosomal Distribution, Gene Duplication and Dual Synteny Analysis of OsLBD Genes

To determine the chromosomal localization of OsLBD genes, we input RGAP IDs in Oryzabase (an integrated Rice Science Database, https://shigen.nig.ac.jp/rice/oryzabase/, accessed on 1 January 2025) to identify and visualize the physical location of each gene on chromosomes. We performed a collinearity analysis to investigate the gene duplication event among OsLBD gene members and their orthologous OsLBD genes (dual synteny) in Arabidopsis and barley species using MCScanX (Multicollinearity Scanning toolkit) with default settings [31]. The results were visualized by using advanced Circos and dual synteny plot tools in TBtools.

### 3.5. Expression Profiling and Identification of Co-Expressed of OsLBD Genes During Whole Life Cycle of Rice

Microarray expression data for rice (*Oryza sativa*) were obtained from the CREP database (http://crep.ncpgr.cn/crep-cgi/home.pl, accessed on 1 January 2025), which provides a comprehensive resource of rice gene expression profiles. The dataset is derived from two elite *indica* cultivars, Zhenshan 97 and Minghui 63 (the parents of the widely cultivated hybrid Shanyou 63), encompassing 39 tissues and organs sampled across the entire life cycle (from germination to mature seeds) of the plant and 24 tissues were used in our study (full tissue list and developmental stages are provided in Table S3) [32]. Seedlings at 3 days after sowing and at the trefoil stage, as well as shoots and roots at the two-tiller stage, were collected from plants cultured hydroponically. For the hormonal treatments included in our analysis, seedlings at the trefoil stage were treated with 100 μM of gibberellin (GA3), auxin (NAA), or cytokinin (kinetin). Samples were collected at multiple time points (5, 15, 30, and 60 min) post-treatment and pooled for each hormone for representing a broad transcriptional response.

Field-collected tissues (leaf, leaf-sheath, stem, flag leaf, palea, lemma, spikelet, endosperm and panicle) were grown under normal agricultural conditions in Wuhan, China. All samples were harvested and immediately frozen in liquid nitrogen, and stored at −70 °C until RNA extraction. Critically, each sample in the database constitutes a biological replicate, with tissues pooled from at least five individual plants. To generate a robust and consolidated expression profile for subsequent analysis, the raw expression values for each sample were averaged from either two or three biological replicates, with the higher-signal value probe selected for genes represented by multiple probe sets (Table S3). This approach is a standard practice for creating a unified expression matrix for large-scale transcriptomic meta-analyses. The final averaged dataset was used for all downstream analyses, including tissue-specific expression profiling, hierarchical clustering, and the construction of co-expression networks.

Co-expression networks were constructed using pairwise Pearson correlation coefficients (r ≥ 0.8) between *OsLBD* genes and all other co-expressed genes, following established protocols [33]. Weighted Gene Co-expression Network Analysis (WGCNA) was implemented using the R V1.73 package (https://rdocumentation.org/packages/WGCNA/versions/1.73) (accessed on 1 January 2025) to identify significantly correlated gene modules. Functional enrichment of co-expressed genes was analyzed via the ShinyGO webserver (http://bioinformatics.sdstate.edu/go/) accessed on 1 January 2025.

Raw signal values were log2 transformed to normalize variance, and the tissue-specific expression patterns of *OsLBD* genes were visualized using TBtools via hierarchical clustering (Euclidean distance, complete linkage) [30]. For data analysis, *OsLBD* genes were classified as differentially expressed in each tissue if they showed significant up-regulation (*p* < 0.05, fold change >2) or down-regulation (*p* < 0.05, fold change < 0.5) relative to seed tissue.

## 4. Discussion

Rice is a vital staple crop for nearly half of the global population and serves as an important model organism for monocot functional genomics research [34,35]. With global population growth and rising living standards projected to increase food demand by 50% by 2050, enhancing rice yield has become an urgent agricultural priority [36]. Addressing this challenge requires substantial improvements in rice production and quality through advanced genetic and molecular approaches [37,38]. A comprehensive understanding of the regulatory networks controlling rice growth and development, particularly those mediated by transcription factors, is therefore critical for devising targeted strategies to improve crop yield and ensure future food security. The *LBD* gene family constitutes a critical group of transcription factors that encode plant-specific proteins characterized by a conserved LOB domain. These proteins play essential roles in regulating diverse biological processes in higher plants, including lateral organ formation, metabolic regulation, and stress responses. Given their functional importance, *LBD* genes have been systematically identified in multiple plant species, including *Arabidopsis* (43 members), maize (44 members), barley (34 members), and wheat (90 members) [14,16,39,40]. Our comprehensive genomic survey identified 35 *OsLBD* genes in the *Oryza sativa ssp. japonica* genome, revealing a larger family than previously reported by Zhao et al. (2023) in rice [24]. But our number differs from a recent study by Sun et al. (2025), which reported 36 members [41]. This discrepancy arises from our stringent filtering criteria, which required the presence of a full or partial LOB domain for inclusion. Our initial search identified 39 candidates, but four genes were excluded due to the absence of this defining domain. Our focused analysis on the 35 domain-containing genes provides a conservative and functionally relevant framework for the core OsLBD family in rice. However, in our study, the expansion appears to have occurred mainly through segmental duplication rather than tandem duplication, with all duplicated genes confined to Class I subclasses [42]. The retention of these duplicated pairs—particularly those exhibiting tissue-specific co-expression, such as LOC_Os08g31080 and LOC_Os09g19950 in stamen tissue—suggests that subfunctionalization has played a key role in preserving these gene copies during evolution. In contrast, the apparent pseudogenization of LOC_Os02g57490 illustrates how redundancy can lead to gene degeneration. These patterns are consistent with the duplication–degeneration–complementation model of gene family evolution [43,44]. The complete absence of duplications among Class II members indicates that distinct evolutionary pressures may act on the two classes, reflecting their potentially different functional roles as transcriptional regulators.

Phylogenetic analysis supported the division of *OsLBD* proteins into Class I and Class II, consistent with the classification established in *Arabidopsis* [20,39]. Class I was further subdivided into five subclasses (IA–IE) based on conserved domain architecture. The remarkable conservation of the zinc-finger-like (CX_2_CX_6_CX_3_C) and leucine-zipper (LX_6_LX_3_LX_6_L) motifs in Class I proteins across both rice and *Arabidopsis* underscores the essential roles of these domains in DNA binding and protein dimerization, whereas the absence of the leucine-zipper (LX_6_LX_3_LX_6_L) motif in Class II members suggests alternative mechanisms of action [45]. The predominantly nuclear localization predicted for all OsLBD proteins aligns with their putative roles as transcription factors, while their generally unstable nature—with only two exceptions—may reflect regulatory mechanisms that control protein turnover in response to developmental or environmental cues.

The chromosomal distribution of *OsLBD* genes displayed distinct patterns, with no genes located on chromosomes 4 and 6, while notable clustering occurred on chromosomes 1 and 3. The presence of gene clusters—particularly the six tandemly arrayed *OsLBD* genes within a 200 kb region on chromosome 1—suggests that localized duplication events may have contributed to the expansion of this gene family. Similar clustered arrangements have been reported in other species, including wheat, cotton, and maize, where such organization may facilitate coordinated gene regulation [14,16].

Gene structure analysis provides valuable insights into the phylogenetic relationships and evolutionary history of gene families. Our analysis revealed that closely related *OsLBD* genes generally share similar structural features, including conserved motif arrangements and comparable exon–intron architectures. Notably, most *OsLBD* genes possess relatively simple genomic structures, containing only one or two exons. This simplicity may confer evolutionary flexibility, enabling rapid neofunctionalization following duplication events [46]. We observed remarkable uniformity in gene structure within certain subclasses, particularly subclass ID, where all members contained a single exon except for one. In contrast, subclasses IA and IIB displayed greater structural heterogeneity, which may reflect differences in evolutionary history and functional specialization. Protein sequence analysis identified four core conserved motifs, with Motif 4—containing the leucine-zipper domain—being exclusive to Class I members. This observation strongly supports our phylogenetic classification and highlights a fundamental structural divergence between Class I and Class II OsLBD proteins [47].

Gene expression is mainly regulated through interactions between transcription factors and their corresponding *cis*-regulatory DNA sequences within promoter regions [48]. Our analysis identified numerous functional motifs in the promoters of *OsLBD* genes, including elements responsive to phytohormones, developmental cues, and environmental stresses. These findings provide valuable insights into the regulatory networks controlling *OsLBD* gene expression. The high abundance of ABRE elements (responsive to abscisic acid) and methyl jasmonate-responsive motifs across both Class I and Class II suggests that these transcription factors may integrate multiple hormonal signals [49,50]. Additionally, the widespread presence of MYB binding sites in most subclasses indicates their involvement in drought response pathways [51]. The specific enrichment of certain elements, such as the light-responsive 3-AF1 motif uniquely found in LOC_Os01g07480, may reflect specialized regulatory roles for individual family members [52,53].

Expression profiling under hormone treatments revealed that OsLBD genes are differentially regulated by auxin, cytokinin, and gibberellin, with most showing downregulation, suggesting a primary role as negative regulators in hormone signaling pathways [54]. Notably, LOC_Os01g66590 was specifically upregulated in response to cytokinin treatment, indicating a potential positive regulatory function in cytokinin-mediated processes [55]. Moreover, the observed expression patterns partially corresponded with the presence of hormone-responsive *cis*-elements in their promoters, supporting the biological relevance of our in silico predictions. Comprehensive expression analysis across the rice life cycle revealed that *OsLBD* genes display highly distinct and often tissue-specific expression patterns. Genes such as LOC_Os08g31080 and LOC_Os09g19950 were specifically upregulated in reproductive tissues, particularly stamens, indicating roles in floral development, whereas broadly expressed genes such as LOC_Os07g40000 may function in general growth regulation. Hierarchical clustering of expression profiles grouped these genes into distinct clusters, with one cluster exhibiting particularly high expression during reproductive stages, suggesting coordinated regulation of panicle and flower development.

Comparative genomic analysis revealed substantially higher collinearity between rice and barley *LBD* genes than between rice and *Arabidopsis*, reflecting the closer phylogenetic relationship within the Poaceae family. The conservation of syntenic blocks containing *LBD* genes in both rice and barley suggests the preservation of important genomic contexts, whereas the limited conservation with *Arabidopsis* highlights the evolutionary divergence between monocots and dicots [56].

The distinct functional specialization of co-expressed *OsLBD* gene modules revealed by our analysis provides valuable insights into the molecular organization of rice developmental and stress response pathways (Figure 10) [26]. The turquoise module, with pollen-specific expression and enrichment of cell wall–related genes—particularly pectin lyase fold and calcium-binding EF-hand domain proteins—strongly suggests a role in pollen tube growth and cell wall remodeling, processes essential for successful fertilization [57]. In contrast, the brown module, exhibiting endosperm-preferential expression and association with lipid transfer and starch metabolism genes, highlights its critical function in nutrient accumulation during seed development, a key determinant of seed quality and viability [58]. The functional partitioning observed in the other modules further highlights the remarkable specialization of these gene networks. The yellow module, enriched for photosynthetic and photoprotective functions, likely reflects activity in photosynthetic tissues or developmental stages with high energy demands [59]. Similarly, the blue module, broadly associated with transcriptional regulation, reproductive development, and cell division, may function as a central coordinator of core biological processes [60]. The green module, related to hormonal and oxidative stress responses, appears to act as an integrator of environmental signals and developmental programs through ROS homeostasis and hormone signaling pathways (Figure 10) [61]. This modular organization of stress-responsive genes may represent an evolutionary adaptation to balance growth and defense mechanisms under fluctuating environmental conditions [62].

While our study provides substantial insights into the *OsLBD* gene family, several key questions remain unresolved. Through comprehensive gene structure analysis, conserved domain characterization, and phylogenetic reconstruction, we classified *OsLBD* members into distinct clades, revealing evolutionary patterns influenced by segmental duplication events. Chromosomal localization and collinearity analyses further highlighted lineage-specific mechanisms of gene family expansion. Future functional characterization of individual *OsLBD* members—particularly those exhibiting reproductive tissue-specific expression or strong hormone responsiveness—will be essential to elucidate their precise roles in rice development. The potential roles of *OsLBD* genes in stress responses, suggested by the abundance of stress-related *cis*-elements in their promoters, require experimental validation under diverse abiotic and biotic stress conditions. Furthermore, the protein–protein and protein–DNA interaction networks regulating *OsLBD* function remain largely uncharacterized. Future studies using yeast two-hybrid and one-hybrid systems, co-immunoprecipitation, ChIP-qPCR, and electrophoretic mobility shift assays could elucidate their underlying molecular mechanisms. The generation of CRISPR-Cas9 knockout lines for key *OsLBD* members would enable direct assessment of their phenotypic impacts, particularly on agronomically important traits such as panicle architecture and grain yield. Overall, the insights gained from this comprehensive analysis provide valuable guidance for targeted rice improvement strategies. The identification of stamen-specific *OsLBD* genes provides potential targets for manipulating reproductive development and improving grain production, while hormone-responsive members could be leveraged to optimize growth under varying environmental conditions. The evolutionary patterns revealed through comparative genomics may guide similar studies in other cereal crops, facilitating cross-species knowledge transfer. By establishing this foundational framework for the *OsLBD* gene family in rice, our work enhances the understanding of OsLBD transcription factor evolution and function in plants.

## 5. Conclusions

Our systematic analysis of the rice *OsLBD* gene family identified 35 members, classified into Class I and Class II. These transcription factors possess conserved DNA-binding domains and display tissue-specific expression, particularly in reproductive organs, indicating roles in developmental processes and stress adaptation. Promoter analysis revealed hormone-responsive elements, while expression profiling demonstrated regulation by auxin and cytokinin pathways. Co-expression network analysis linked specific *OsLBD* members to reproductive development, with specialized functions in pollen cell wall synthesis and endosperm glycogen biosynthesis. This study provides a molecular framework for leveraging *OsLBD* genes in rice and other agronomic crops through targeted genetic improvement strategies.

## Figures and Tables

**Table 1:** Characteristics of *OsLBD* transcription factor family.

Gene IDs	AA Length	MW (Da)	pI	Negative Charges	Positive Charges	Instability Index	Stability	Aliphatic Index	GRAVY	Cellular Location
LOC_Os01g03890	307	32,379.55	6.89	30	29	52.62	Unstable	76.42	−0.297	Nucleus
LOC_Os01g07480	251	25,728.12	7.61	18	19	51.93	Unstable	78.65	0.003	Nucleus
LOC_Os01g14030	249	26,091.37	9.72	18	31	45.7	Unstable	67.27	−0.321	Nucleus
LOC_Os01g32770	330	35,019.79	5.36	48	32	54.31	Unstable	77.27	−0.399	Nucleus
LOC_Os01g39040	356	38,510.31	4.9	53	38	77.89	Unstable	77.89	−0.396	Nucleus
LOC_Os01g39070	125	13,306.08	6.26	13	12	68.08	Unstable	68.16	−0.425	Nucleus
LOC_Os01g39150	251	26,857.04	5.69	30	23	75.7	Unstable	62.71	−0.649	Nucleus
LOC_Os01g39160	171	17,865.16	5.25	22	17	46.31	Unstable	72.75	−0.127	Nucleus
LOC_Os01g39180	321	35,174.2	9.45	29	39	64.75	Unstable	77.32	−0.328	Nucleus
LOC_Os01g39220	139	14,864.02	6.03	13	11	69.05	Unstable	77.41	−0.192	Nucleus
LOC_Os01g56530	245	25,730.04	7.72	22	23	50.79	Unstable	82.12	−0.147	Nucleus
LOC_Os01g60960	251	26,493.01	6.45	26	24	54.8	Unstable	76.29	−0.173	Nucleus
LOC_Os01g66590	269	27,621.93	8.17	17	19	39.38	**Stable**	69.89	−0.08	Nucleus
LOC_Os02g57490	216	22,457.64	8.09	15	17	53.54	Unstable	78.43	0.103	Nucleus
LOC_Os03g05500	185	19,631.01	6.05	14	12	60.74	Unstable	66.65	−0.186	Nucleus
LOC_Os03g05510	259	26,736.57	5.96	21	14	43.6	Unstable	65.52	−0.085	Nucleus
LOC_Os03g14270	275	28,252.75	8.27	15	17	83.07	Unstable	67.53	−0.321	Nucleus
LOC_Os03g17810	239	25,556.79	6.06	23	20	62.94	Unstable	76.4	−0.241	Nucleus
LOC_Os03g33090	204	21,037.1	8.41	16	19	50.07	Unstable	81.37	0.042	Nucleus
LOC_Os03g41330	232	23618.55	6.05	19	17	49.15	Unstable	81.34	0.023	Nucleus
LOC_Os03g41600	307	33,077.8	6.65	25	20	58.81	Unstable	64.01	−0.577	Nucleus
LOC_Os03g45750	214	22,012.96	9.06	15	21	56.78	Unstable	70.75	−0.131	Nucleus
LOC_Os03g57670	171	18,322.4	7.63	17	18	65.46	Unstable	56.2	−0.458	Nucleus
LOC_Os05g03160	145	14,935.02	4.96	13	7	46.9	Unstable	85.86	0.166	Nucleus
LOC_Os05g07270	207	21,567.58	8.98	12	17	63.58	Unstable	83.19	0.11	Nucleus
LOC_Os05g27980	200	21,105.84	6.28	17	14	53.5	Unstable	78.2	−0.145	Nucleus
LOC_Os05g34450	279	28,044.35	8.23	16	18	41.59	Unstable	71.08	−0.004	Nucleus
LOC_Os07g40000	226	23,216.21	7.54	19	19	56.33	Unstable	69.16	−0.115	Nucleus
LOC_Os08g06659	196	21,283.44	4.62	36	18	81.05	Unstable	54.49	−0.88	Nucleus
LOC_Os08g31080	456	48,653.41	4.69	68	31	38.04	**Stable**	72.08	−0.474	Nucleus
LOC_Os08g44940	215	23,239.17	5.85	17	12	48.33	Unstable	67.26	−0.16	Nucleus
LOC_Os09g19950	362	37,637.55	5.27	37	22	46.48	Unstable	65.19	−0.397	Nucleus
LOC_Os10g07510	269	27,974.12	6.41	20	16	66.24	Unstable	67.55	−0.336	Nucleus
LOC_Os11g01550	156	17,404.87	6.42	14	12	57.14	Unstable	77.05	−0.254	Nucleus
LOC_Os12g01550	156	17,404.87	6.42	14	12	57.14	Unstable	77.05	−0.254	Nucleus

Abbreviations: AA, amino acid; MW, molecular weight; pI, isoelectric point; GRAVY, grand average of hydropathicity. Only two proteins (bold) were predicted as stable. The proteins containing the instability index threshold value of more than 40 were considered unstable.

## Data Availability

All data generated or analyzed during this study are included in this published article and its Supplementary Information Files. The raw data for expression analysis can provided by the authors on request.

## Supplementary Materials

The following supporting information can be downloaded at https://www.mdpi.com/article/10.3390/plants14233596/s1, Table S1. a. Identification of cis-elements in 2-kb promoter regions of OsLBD genes. b. The number of cis-elements on putative promoters of OsLBD genes. Table S2. Results of differential expression analysis under three phytohormone (NAA, GA3 and KT) treatments in Minghui 63. Table S3. The detailed information of samples used in microarry analysis from CREP database. Table S4. Expression pattern of duplicated gene pairs. Table S5. Results of differential expression analysis using seed as reference in Minghui 63. Table S6. OsLBD genes and their co-expression modules in WGCNA analysis.
